# Disrupted prefrontal functional connectivity during post-stress adaption in high ruminators

**DOI:** 10.1038/s41598-018-33777-1

**Published:** 2018-10-22

**Authors:** David Rosenbaum, Paula Hilsendegen, Mara Thomas, Florian B. Haeussinger, Hans-Christoph Nuerk, Andreas J. Fallgatter, Vanessa Nieratschker, Ann-Christine Ehlis, Florian G. Metzger

**Affiliations:** 1 Department of Psychiatry and Psychotherapy, University Hospital of Tuebingen, Tuebingen, Germany; 2 Center of Integrative Neuroscience (CIN), Cluster of Excellence, University of Tuebingen, Tuebingen, Germany; 3 Department of Psychology, University of Tuebingen, Tuebingen, Germany; 4 LEAD Graduate School & Research Network, University of Tuebingen, Tuebingen, Germany; 5 Leibniz-Institut für Wissensmedien, Tuebingen, Germany; 6 Geriatric Center, University Hospital Tuebingen, Tuebingen, Germany

## Abstract

Rumination is a perseverative thinking style that is associated with adverse mental and physical health. Stressful situations have been considered as a trigger for this kind of thinking. Until today, there are mixed findings with respect to the relations of functional connectivity (FC) and rumination. The study at hand aimed to investigate, in how far high and low ruminators would show elevated levels of state rumination after a stress induction and if these changes would show corresponding changes in FC in the cognitive control network (CCN) and dorsal attention network (DAN). 23 high and 22 low trait ruminators underwent resting-state measurements before and after a stress induction with the Trier Social Stress Test (TSST). Changes in rsFC through the TSST were measured with functional near-infrared spectroscopy within and between regions of the CCN. Stress successfully induced state rumination in both groups but stronger in the high trait ruminators. High trait ruminators showed elevated FC within the CCN at baseline, but attenuated increase in FC following the TSST. Increases in FC correlated negatively with state rumination. A lack of FC reactivity within the CCN in high ruminators might reflect reduced network integration between brain regions necessary for emotion regulation and cognitive control.

## Introduction

The tendency to ruminate about negative thought content has been shown to be related to a variety of adverse consequences^1^. Rumination can be defined as self-referential persistent repetitive and rather pessimistic thinking style about the past, ones mistakes or shortcomings, with little or no change and goal-orientation^2^. In the case of mental disorders, rumination is related to the onset, duration and reoccurrence of depressive episodes^1^ and to the maintenance of social phobia^3^. On a neural level, rumination has been shown to be related to various functional alterations in different networks, both regarding activation patterns^4–9^ and functional connectivity (FC)^10–12^. Regarding the default mode network (DMN), rumination has been linked to elevated FC between the subgenual anterior cingulate cortex and parts of the DMN, including parts of the posterior cingulate cortex^4^. Furthermore, hypo-connectivity within frontoparietal control networks, within the dorsal attention network (DAN) and hyper-connectivity between the cognitive control network (CCN) and the DMN have been observed^12–14^. However, some studies also showed higher FC within the CCN^15,16^, and reduced FC in inter-hemispheric FC indices^17–20^. So far, most resting-state studies that tried to assess the relationship between FC and rumination used either non-inductive measurements, by correlating the rumination response scale (RRS) with FC during resting-state, or by inducing rumination through biographical induction tasks^21^. Since the RRS is a trait-like measure, recently certain attempts have been made to develop state rumination questionnaires^13,22^. In contrast to trait measures, state rumination questionnaires aim to assess the current state of the construct (which only correlates moderate with the trait), e.g. during a neurophysiological resting state measurement. Also, beside biographical induction methods, indirect induction methods through negative mood inductions have been used^23–25^. Since some theories propose a special role for stressful life events as rumination-eliciting situations^1^, attempts have also been made to induce rumination via stress induction techniques^26–29^ and to measure the influence of rumination on the stress response^28,30^. Indeed, in different studies state rumination has been induced through social stress^27–29^ and rumination clearly has an effect on the stress response. Recent review and meta-analytic data on the physiological effects of rumination showed that rumination is associated with higher systolic (g = 0.45) and diastolic (g = 0.51) blood pressure, higher cortisol (g = 0.32–0.36), heart rate (g = 0.20–0.28) and lower heart-rate variability (g = 0.15–0.27)^31^. Following stress induction, rumination has effects on the cortisol response in terms of a reduced decline^32,33^. This effect might be more strongly related to state rumination as compared to trait rumination^27^.

There is a large body of literature on the issue of stress effects on brain activity^34^ and connectivity (e.g., see the review by van Oort^35^). With respect to the effects of stress on resting state FC directly after the stress-induction four studies exist^36–39^. In three of these studies a seed based approach has been used, which consistently yielded the result of increased FC between the amygdala and DMN related brain areas such as the hippocampus and parahippocampal gyrus^35,37–39^. Furthermore, the study of Meron-Katz (2016) used a large scale network approach which investigated FC changes through stress between different brain areas. Following stress, the authors reported increased absolute resting state FC and more concretely increased thalamo-cortical FC, including the frontal, temporal and parietal lobes^36^. However, also decreased FC between cross-hemispherical temporo-parietal areas has been reported in this study.

In the current study, we sought to investigate changes in resting-state FC in low and high trait ruminators following a stress induction via the Trier Social Stress Test (TSST). Additionally, we assessed quantitative rumination state-variables to investigate in how far social stress elevates ruminative responses following the stress induction. In our primary analysis of the same sample, we already showed that high ruminators show reduced cortical activation during the performance of the TSST in comparison to low ruminators^40^. Additionally, cortical reactivity through the TSST mediated group differences in negative affect and state rumination following the TSST procedure. Since measures of functional connectivity give additional information about regional integration and segregation during information processing, in the present work, we investigated changes in resting-state FC through the TSST in high and low ruminators.

We hypothesized that the stress induction would lead to higher FC within the CCN and the DAN and that these changes would still be present in a resting-state measure following the TSST (hypothesis 1). From our previous investigations, we expected that high ruminators in contrast to low ruminators would show higher FC in the CCN before the TSST (hypothesis 2).

Further, from prior data on differences in FC reactivity between depressed and non-depressed subjects^12^, we hypothesized that the high ruminators would show attenuated FC in the Cognitive Control Network (CCN) and DAN following the TSST (hypothesis 3). We further explored the correlations between state rumination, negative affect and increases in FC.

## Methods

### Participants

This study was approved by the ethics committee at the University Hospital and University of Tuebingen. All used methods and procedures in this study were in accordance to the current guidelines of the World Medical Associations Declaration of Helsinki. 45 subjects – 23 high and 22 low ruminators – were recruited at the University of Tübingen according to their total RRS score. In this study, the 22-item RRS version was used^41^. Further, before participation subjects were screened with the Structural Clinical Interview for DSM IV (SCID) to screen for possible prior diagnosis^42^. Exclusion criteria included left-handedness, medication (with exception for contraceptive medication), medical conditions that influence the stress response and cigarette smoking (more than three cigarettes per week). The sample was recruited out of a sample of 400 subjects that completed an online assessment of the RRS. High ruminators had to have a mean RRS score higher than 2.36 (PR > 65) and low ruminators had to have an RRS score lower than 1.9 (PR < 27). The cut-off definition was based on RRS scores of clinical samples of patients with Major Depressive Disorder in the University Hospital of Tübingen^40^. Low ruminators were on average age 22 years old (86% female). Their mean BDI-II score was 1.9 which implies the absence of depressive symptoms. The high rumination group was 79% female and was on average 22 years of age. The mean BDI was 8.5, which also implies the absence of clinically-relevant symptoms. However, both groups differed significantly with respect to their BDI scores, indicating subclinical symptoms in the high ruminators (see Table 1).

**Table 1:** Demographic variables of the high and low rumiantion group. BDI = Beck Depression Inventory, RRS = Rumination Response Scale, NA = negative affect from the Positive and Negative Affect Schedule.

Variable	Low Ruminators (n = 22)		High Ruminators (n = 23)			
	mean	SD	Mean	SD	t/χ²	P
Age (years)	22.3	3.88	21.69	2.68	t_(43)_ < 1	p > 0.1
Percent of female participants	86%		79%		χ²_(1)_ = 0.5	p > 0.1
BDI score	1.9	2.25	8.5	5.80	t_(43)_ = 4.99	p < 0.001
RRS score	1.5	0.21	2.6	0.17	t_(43)_ = 19.32	p < 0.001
hours spent ruminating per day	0.25	0.38	0.55	0.55	t_(43)_ = −2.105	p < 0.05
State-rumination post TSST	1.44	0.43	2.45	1.07	t_(43)_ = 4.12	p < 0.001
NA post TSST	16.81	5.377	23.61	9.03	t_(43)_ = 3.05	p < 0.01
Qualitatively reported rumination during post- stress resting-state	2.05	2.13	4.0	3.12	t_(43)_ = −2.42	p < 0.05
Rumination score (Interview)	7.50	3.0	10.2	2.95	t_(43)_ = −2.96	p < 0.01

The pre-experimental assessment of ruminative behavior via interview (see supplementary material) indicated significant differences between the groups in the following dimensions: more dwelling thoughts (χ²_(2)_ = 5.8, p < 0.05), higher persistence (χ²_(3)_ = 5.8, p < 0.001), higher rumination-associated guilt (χ²_(1)_ = 7.9, p < 0.01) and shame (χ²_(2)_ = 7.9, p < 0.05), higher rumination-associated hopelessness (χ²_(2)_ = 14.96, p < 0.001), more dwelling on “why-questions” (χ²_(3)_ = 9.67, p < 0.05) and higher subjective impairment though rumination (χ²_(2)_ = 18.18, p < 0.001) in the high ruminators.

### Procedures

At the day of the measurement, all subjects gave written informed consent and completed an interview in which basic (demographic) variables and rumination-related behavior were assessed. Afterwards, subjects were brought to the NIRS laboratory were they underwent a 7 minute resting-state measurement with open eyes. Then participants performed two control tasks (reading numbers and doing subtractions) with 12 minutes duration before completing the TSST with approximately 16 minutes duration. During the TSST the committee (a female and male judge) entered the room and took place in front of the subjects. According to the TSST protocol^43^ the participants were told that they applied for an job and had a 5 min preparation phase (anticipatory stress phase) before performing a 5 min free speech about their personal strengths and qualifications. During the free speech, the subjects stood in front of the non-verbal neutral and emotional non-responsive TSST committee and were videotaped. In a third phase, subjects were asked to perform a 6 min arithmetic task (arithmetic stress challenge). Again, subjects had to do subtractions (subtracting the number 13 from different starting points between 1026 and 1014) but were interrupted by a committee member if they made an error. Further subjects were asked to perform better and faster from time to time (see^40^). After completion of the TSST, a second resting-state measurement was performed. Directly following each resting-state measurement, subjects completed two resting-state questionnaires that were adapted from the Amsterdam Resting-State Questionnaire^44^ to assess state rumination. After the second resting-state, qualitative self-report forms were used to assess cognitive reactions (e.g., rumination) after the stress induction. The self-report forms were quantified by the procedure used by Shull *et al*. (2016) in which each sentence is rated with respect to ruminative content^28^.

**Figure 1:**
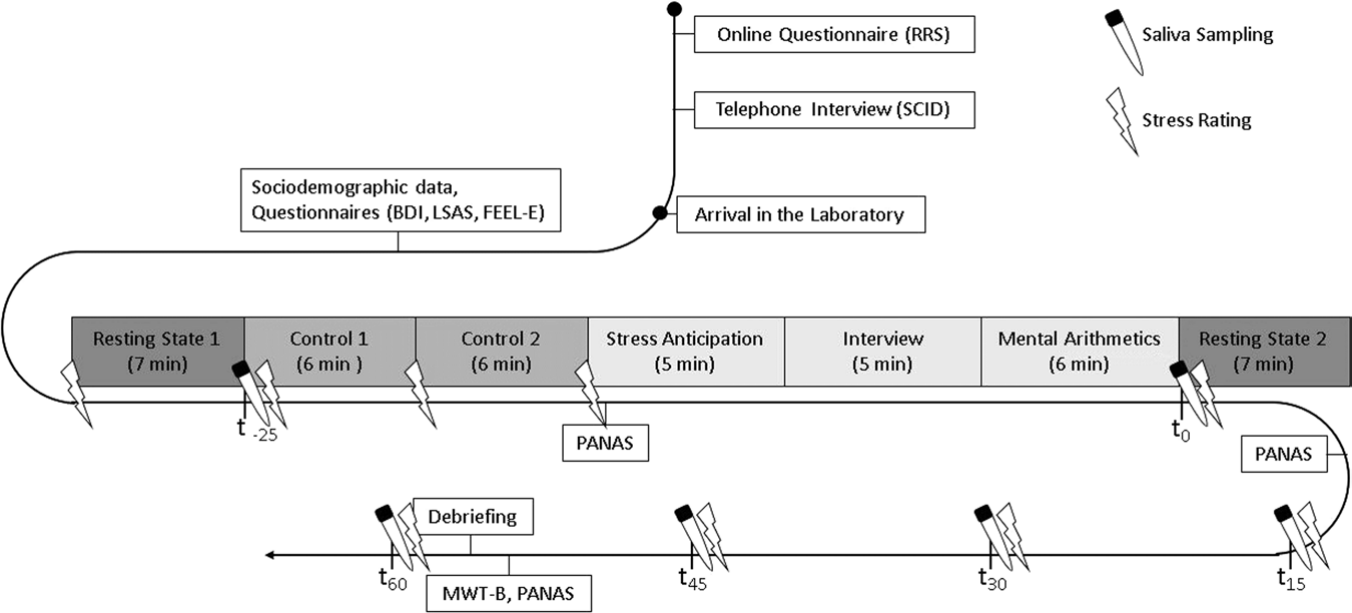
Experimental procedures of the whole experiment.

Salivary-samples were taken before the experimental procedure and up to one hour after completion of the TSST. Additionally, subjective stress ratings and heart rate measures were assessed during the different parts of the experimental procedure (see Fig. 1).

### Functional near-infrared spectroscopy (fNIRS)

Hemodynamic fluctuations were assessed with a continuous wave, multichannel NIRS system (ETG-4000 Optical Topography System; Hitachi Medical Co.,Japan) with a temporal resolution of 10 Hz. In total, three probesets were used including two frontal and one parietal measurement array. Optodes were placed on a combined electrode Easycap with sponge rings for additional fixation. The system consisted of 46 channels (see Table 2).

**Table 2:** Channels of the used probesets and corresponding brain areas.

Brain area	Probeset	
	Probeset: left frontal	Probeset: right frontal
Retrosubicular area	1	14, 16
Dorsolateral Prefrontal Cortex	5, 10, 11, 12	15, 20, 23, 24
Temporopolar Area	2	13
Subcentral Area	3	17
Pre-Motor and Supplementary Motor Cortex	8	22
Pars Opercularis	6	19
Pars Triangularis	4, 7, 9	18, 21
	Probeset: parietal	
Somatosensory Association Cortex	25, 26, 27, 28, 30, 31, 32, 34, 35, 36, 37	
V3	38, 39, 40, 41, 43, 44, 45, 46	
Angular Gyrus	42	
Supramarginal Gyrus	29, 33	

Data was analyzed using MATLAB R2017a (MathWorks Inc, Natick, USA). Preprocessing included a first bandpass filter (0.1–0.001 Hz), movement artefact reduction by the algorithm of Cui *et al*.^45,46^ and interpolation of single noisy channels. In 16 subjects, channels had to be interpolated. However, no more than three channels were interpolated per measurement in any of the subjects. Afterwards, clenching artefacts were reduced with independent component analysis and a second bandpass filtering (0.1–0.01 Hz) was performed. To reduce global artefacts, a spatial gaussian kernel filter^47^ with a standard deviation of σ = 50 was used. We used a standard deviation of σ = 50 as this yielded the best results in terms of reduction of the global signal without inducing artificial negative activation. FC measures were computed by Fisher’s z-transformation of Pearson coefficients with a zero time-lag. Brain Net Figures were plotted with the MATLAB package *BrainNet Viewer*^48^.

### Data Analysis

We analyzed differences between high and low ruminators in their FC changes through the stress induction. Data with respect to hemodynamic responses during the TSST and the control conditions are reported in a separate analysis since the both project parts are independent from each other^40^. Briefly, our results concerning the TSST showed that subjects showed higher blood oxygenation during the TSST as compared to the control conditions in ROIs of the CCN. Further, high ruminators showed reduced reactivity in the right IFG during the stressful task conditions. On behavioral subscales the primary analysis showed significant within-between subject interactions of time by group in state rumination and negative affect, indicating higher increases of both parameters in high trait-ruminators. We observed no difference between high and low ruminators in cortisol responses and heart rate measures. A graphical summary of the results can be seen in the Supplementary Figs S1–S5. In the following analysis, we focus on changes (from pre- to post-test) in resting-state FC in high and low ruminators due to the stress induction. In contrast to our primary analysis, this follow-up study informs about the variability due to social stress in network coupling in high and low ruminators during resting-state, while the primary analysis focused on blood oxygenation of predefined ROIs.

**Figure 2:**
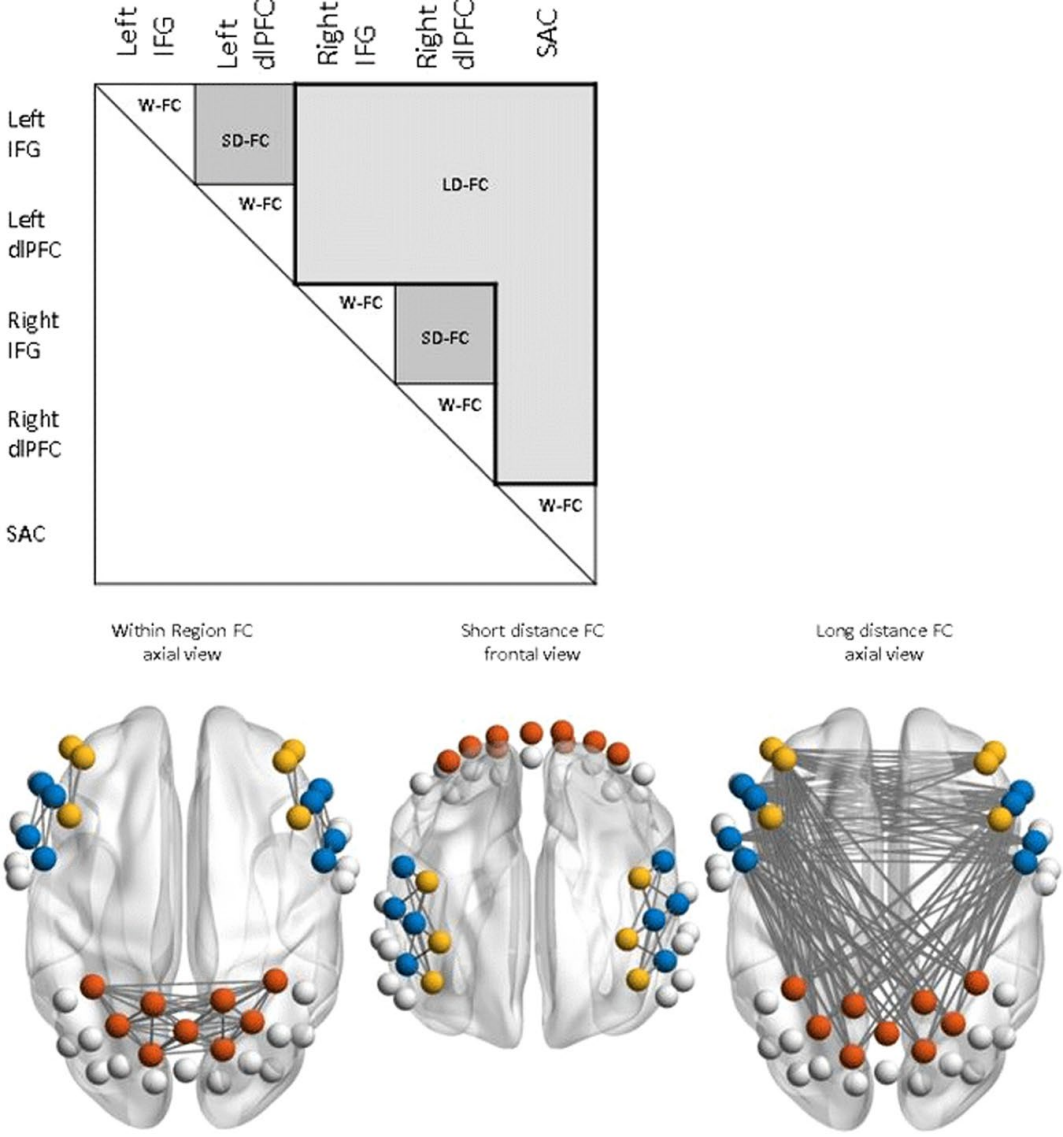
Definition of regions of interest in the analysis and the corresponding within, short-distance and long-distance region connections that were analyzed. The figure was generated with the BrainNetViewer toolbox^48^.

To account for the problem of multiple testing, we investigated the average FC differences between and within pre-defined region-specific nodes (see Fig. 2). As we were interested in the CCN and DAN, we investigated FC between and within the regions of the bilateral dorsolateral prefrontal cortex (dlPFC), inferior frontal gyrus (IFG) and somatosensory association cortex (SAC). As Zhu *et al*.^14^, we separated these connections into within-region FC (within each region), short-distance FC (between the ipsilateral IFG and dlPFC) and long-distance FC (between contralateral dlPFC and IFG regions, frontal regions and superior parietal lobule). For each of these connections were performed a mixed repeated measurements ANOVA with the factors group (high vs. low ruminators) and time (pre-stress vs. post-stress). Correction for multiple comparisons was done by the procedure of Armitage-Parmar at an significance level of α = 0.05^49^. All described results are corrected if not stated otherwise.

## Results

### Behavioral

The quantitative analysis of the qualitative post-stress reports revealed that the high ruminators reported ruminative content more often (on average four sentences with ruminative content vs. two) (t_(43)_ = 2.43, p < 0.05, d = 0.72). With respect to different dimensions of rumination, 54% rehearsed their bad performance, 28% speculated about negative causes or consequences, 39% focused on their negative affect and 59% showed some sort of reflective rumination or cognitive problem solving. Please note that subjects could show more than one dimension in their reports (e.g. first rehearsing bad performance and secondly reflective rumination). Groups differed in the dimension speculating about negative consequences (χ²_(1)_ = 4.87, p < 0.05), with more subjects in the high ruminators (44%) reporting speculations about negative consequences than in the low ruminators (14%). Further, only four subjects (8.9%) reported aggressive impulses towards the TSST committee, while 15 subjects (33.33%) reported feelings of personal failure. Groups did not differ with respect to these qualitative data.

Also, high ruminators showed higher state rumination in general as indicated by the ARSQ state rumination score (t_pre(43)_ = 4.91,p < 0.001, d = 1.45; t_post(43)_ = 4.18,p < 0.001, d = 1.23) and a higher increase in state rumination from pre-TSST to post-TSST resting-state measurements (t_(43)_ = 2.15,p < 0.05, d = 0.82) (see Fig. S1). Measures of heart rate, cortisol and subjective stress ratings (see Figs S2–S4) were significantly influenced by the stress induction as expected and are reported in our previous article on the topic. Further, with respect to negative affect, we observed a significant higher increase in the high ruminators following the stress induction as compared to the low ruminators (see Fig. S5)^40^.

### FC

**Figure 3:**
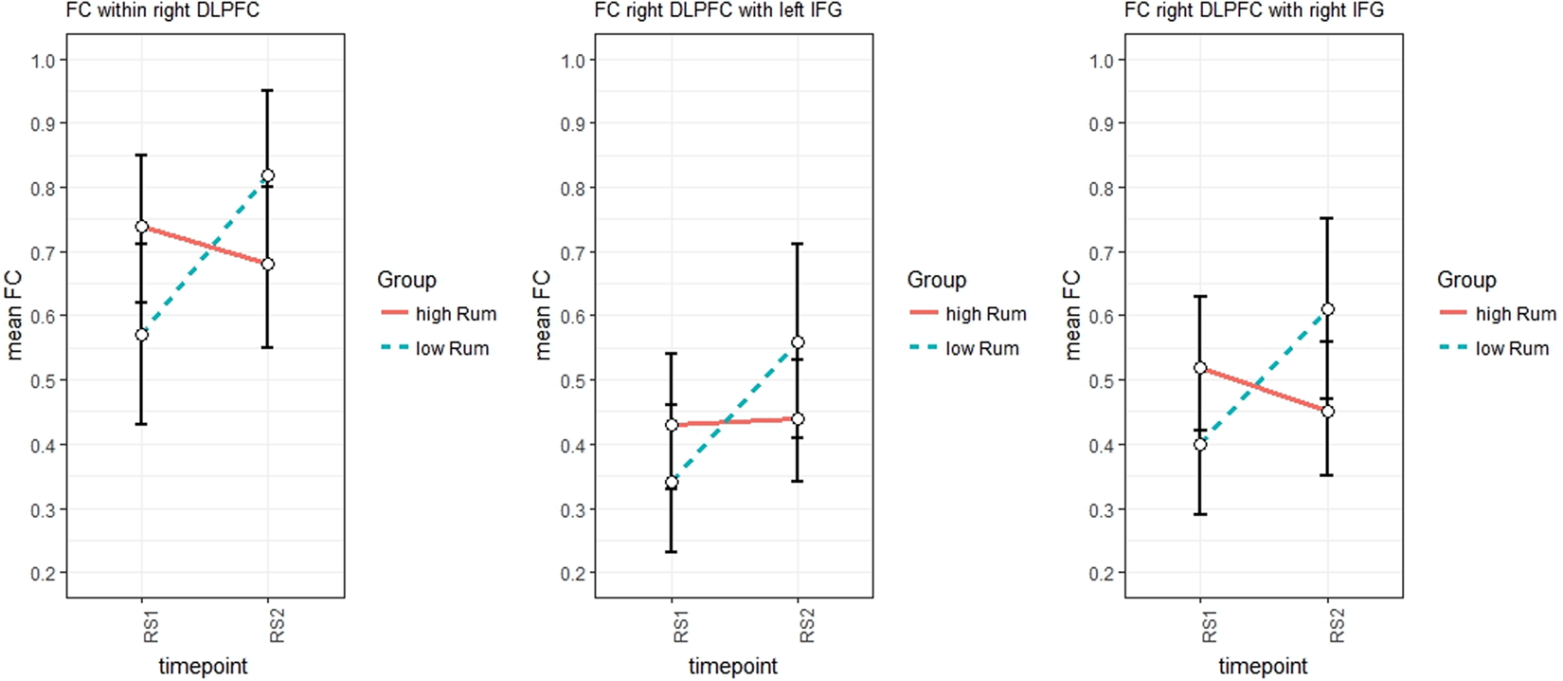
Displaying the disordinal interaction of the time by group effect in three different connections.

Analysis of within-region FC revealed a significant time by group interaction for the right dlPFC (F_(1,43)_ = 8.552, p < 0.01, η² = 0.16) and a marginally significant interaction in the right IFG (F_(1,43)_ = 6.34, p < 0.1, η² = 0.13). Post hoc analysis revealed that this disordinal interaction (see Fig. 3) was driven by a significantly higher increase through the stress induction in the low ruminators (right dlPFC: t_(43)_ = 2.924, p < 0.01, d = 0.87; right IFG: t_(43)_ = 2.51, p < 0.05, d = 0.74) (hypothesis 1), but a significantly higher FC in the high ruminators within the regions before the stress induction (right dlPFC: t_(43)_ = 1.962, p < 0.1, d = 0.58; right IFG: t_(43)_ = 2.54, p < 0.05, d = 0.75) (hypothesis 2).

**Figure 4:**
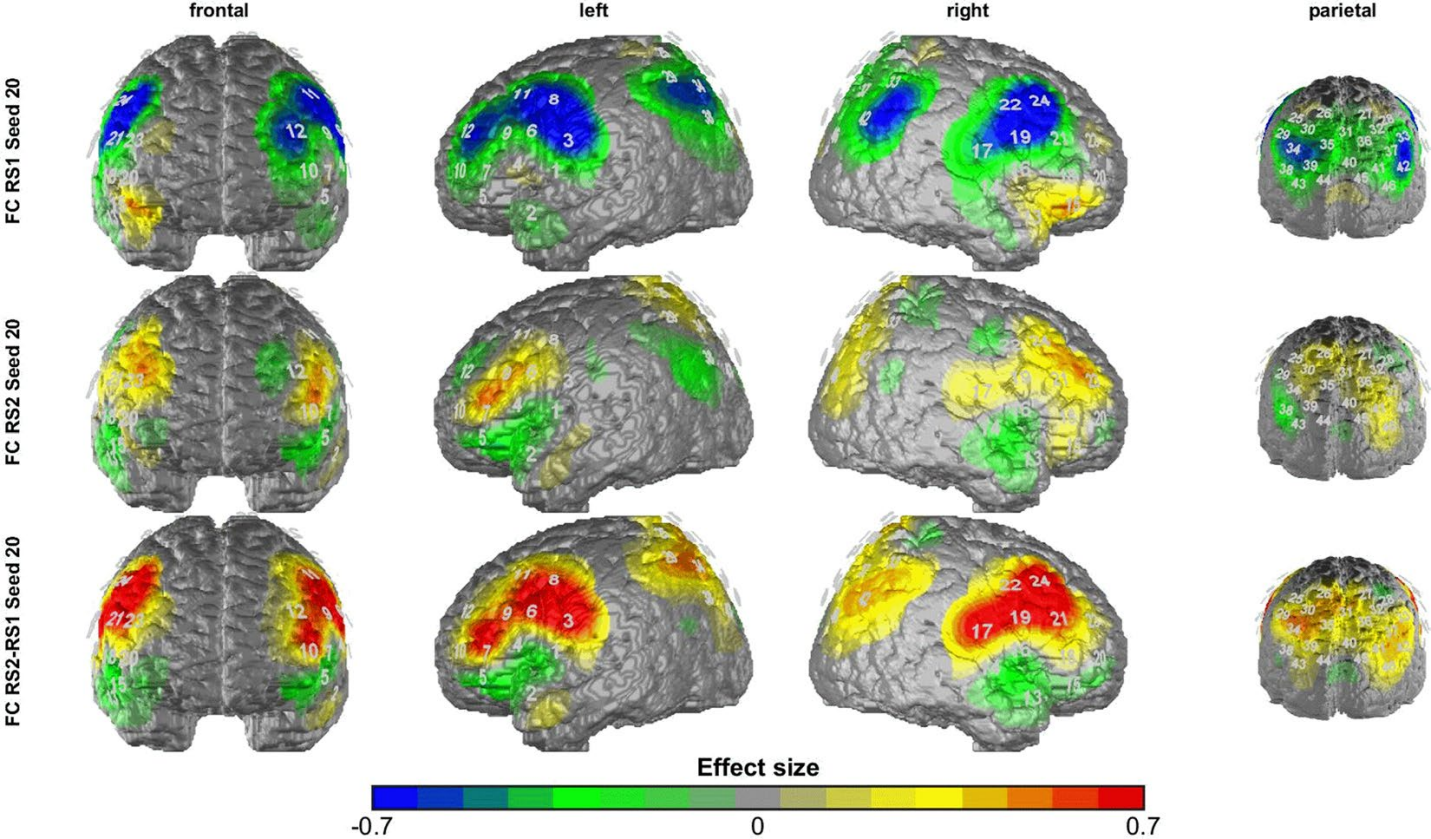
FC differences to a seed region in the right dlPFC between low and high-ruminators for resting-state pre TSST (upper row), post TSST (middle row) and the increase in FC through the TSST (lower row). Warm colors indicate higher FC values/increases for the low ruminators; cold colors indicate higher FC values/increases for the high ruminators.

Accordingly, we found a time by group interaction for the short-distance FC between right dlPFC and right IFG (F_(1,43)_ = 12.981, p < 0.001, η² = 0.231). As for within-region FC, post hoc analysis indicated a higher increase in the low ruminators in FC between the right dlPFC and right IFG following the stress induction (t_(43)_ = 3.59, p < 0.001, d = 1.07) (see Fig. 4) (hypothesis 1).

For long-distance FC, we found a significant main effect for time regarding the FC between right dlPFC and SAC (F_(1,43)_ = 4.26, p < 0.05, η² = 0.09) reflecting a significant increase in FC over the course of the experiment. Also, a significant time by group interaction was found for the coupling of the right dlPFC with the left IFG (F_(1,43)_ = 6.344, p < 0.05, η² = 0.13). Again, increases in FC were higher for the low trait-ruminators (t_(43)_ = 2.52, p < 0.05, d = 0.75) (hypothesis 1).

In a final explorative analysis we also found correlations between FC measures and behavioral measures. Like in our analysis of cortical activation^40^, we investigated the relationship between negative affect, state rumination and increases in FC through the TSST. Significant (but not corrected for multiple comparisons) negative associations were found in FC increases between the right dlPFC and IFG and post-stress negative affect (r_(43)_ = −0.30, p_uncorr_ < 0.05) as well as state rumination (r_(43)_ = −0.29, p_uncorr_ < 0.05), indicating lower post-stress rumination and negative affect in subjects that showed increases in functional integration between the right dlPFC and IFG through the TSST (see Figs S6 and S7). However, this effect was mainly driven by the group differences in post-stress state rumination and FC increases, since the effect was no longer present when correlations were computed for both groups separately. No correlations between FC measures and state rumination were found at pre-TSST.

## Discussion

The aim of this study was to investigate differences in FC between high and low ruminators in the CCN and DAN before and after stress-induced rumination. From our previous investigations, we expected that the high ruminators would show a pattern of elevated FC within the CCN before the stress induction. However, with respect to stress-related FC alterations in this network, we expected high ruminators to be less influenced by the stress induction. It remained an open question if these changes would co-vary with changes in state rumination.

Additionally to the already reported higher reactivity in state rumination and negative affect, we also found higher levels of reported ruminative content during the post-stress resting-state measurement. As indicated by the analysis of sub-dimensions of rumination, this effect was mainly driven by the dwelling on negative consequences and causes of the stress task (e.g. “How did I look like during the testing”, “What did the examiner thought about me and my performance”, “Hopefully I do not meet them again”, “In the future I could fail again in similar situations”, “I thought about other situations in which I failed”). Interestingly, only a few participants reported feelings of anger in their self-report forms, while most subjects reported feelings associated with personal failure like shame or guilt. Regarding the exact nature of the post-stress rumination, this result suggests that it was particularly induced by self-relevant cognitive processes (e.g. regarding the own performance) and related feelings (e.g. shame), which is in line with research that links rumination to such social emotions^50–52^. Although others reported links between rumination and anger^53^, we only found few reported aggressions following the TSST. This might be due to the TSST per se, in which the committee stays non-responsive and neutral, which in turn might foster self-related attributions, rather than situational attributions. Further, it might be a result of timing, since the self-report forms were filled out a few minutes after the TSST. In the emotional aftermath of the experiment, anger about the examiners might have occurred after subjects left the institute.

As reported in our previous article, we did not observe differences between the groups in heart rate or cortisol, which is in line with the work of Ali *et al*. (2017), showing a dissociation of the emotional and affective experience of stress in a study with dexamethasone suppression^54^.

In line with the analysis of cortical activation in this sample while performing the TSST, time by group interactions of the FC measures were found in relevant prefrontal areas for cognitive and attentional control. Our data suggests that the right dlPFC plays a particularly important role in the networks affected by rumination since the region showed aberrant within- and between-region FC in short- and long-range connections with the bilateral inferior prefrontal gyri. However, all of the results showed disordinal interactions of the time-related changes in FC, indicating higher FC in the high ruminators before the TSST and a reduced increase in functional integration through the stress induction. Out of these FC measures, only reactivity scores showed significant negative correlations with state rumination measures after the TSST.

Interestingly, the increase in FC through the TSST in the low ruminators fits well with the current opinion that the CCN is especially active in the aftermath (but not acute phase) of stress^55^ and might reflect effective coping. Indeed, Quaedflieg *et al*. (2015) found higher FC between the left dlPFC and the amygdala in the recovery phase of a stress induction in cortisol non-responders. Additionally, within this study FC between the amygdala and left dlPFC immediately after stress was negatively associated with subjective stress ratings^37^. With respect to the high ruminators, the present findings confirm previous reports of higher FC in the CCN in high ruminators and depressed subjects in non-influenced settings^15,16,56^. However, with respect to the absent increase in FC in high ruminators the results also question in how far these differences reflect state ruminative processes: While state rumination increased in both groups, but more strongly in the high ruminators, increases in FC were only found in the low ruminators. From the data at hand, it is much more likely that the reduced increases in FC in the high ruminators might reflect a reduced ability to adapt to the stress situation which leads to higher negative affect and higher state rumination following the TSST, as reflected by a negative correlation of FC reactivity and post-TSST state rumination and affect. Indeed, others reported increased effective connectivity within CCN regions like the DLPFC and inferior parietal lobule, in subjects during forgiveness to imagined social scenarios^57^. Nonetheless, with respect to our data, this reduced capability of adaption might indeed be influenced by rumination. For example, the higher FC in the high ruminators during the first resting-state measurement might be a result of long-lasting allostatic changes due to high rumination and higher chronic stress levels. These elevated levels of baseline FC might result in a ceiling effect that prevents further increases in FC in the high ruminators. Indeed, in a current study McGirr *et al*. (2017) found elevated global levels of glutamateric FC within a mouse model of depression after exposure to chronic stress. Additionally these effects were reversed by a treatment with ketamine^58^.

Further, the main regions that deviated between high and low ruminators – dlPFC and IFG – have previously been shown to be relevant for successful inhibition, attentional control and emotion regulation^59^. Reduced FC between these areas after stress induction might therefore reflect the reduced capacity of high ruminators to successfully activate a network relevant for information processing during emotion regulation^60,61^, which may lead to the observed pattern of higher negative affect in this subject group following the stress induction.

Interestingly, the results of the FC analysis and previous amplitude analysis of this sample^40^ complement each other. In the same sample, we found reduced cortical activation of high ruminators in response to the TSST challenge in the right IFG and right dlPFC. The same regions showed attenuated increases in FC in the high ruminators following the stress induction, which leads to the conclusion that the prefrontal parts of the CCN show reduced cortical reactivity and task-related network integration in high ruminators. On the other hand, low ruminators were not only able to activate frontal cortical areas more strongly during stress, but also showed higher network integration at resting-state following the stress induction. It will be an endeavor of future research to build models that integrate those different measures of cortical functioning.

## Limitations

Some limitations with respect to the article at hand have to be considered. Firstly, we used fNIRS to assess FC. While the method allows to measure cortical hemodynamics in rather natural settings, its resolution in space is restricted to a rather wide area (3 cm) and only cortical parts of the brain can be assessed. Further, due to a limited number of optodes, only parts of the cortex are measured. It is clearly possible that other areas of the cortex – such as the medial prefrontal cortex – may have shown an increase in FC in the high ruminators that could not be measured with the reported measurement setting. Further, with respect to the research design, we were interested in differences between high and low ruminators. For economic reasons, we did not use active control groups that were not stressed. However, from previous data, we would not expect changes in FC between different resting state measures in such a non-interventional control group^62–64^. With respect to the chosen indirect induction of ruminative processes, it has to be mentioned that the stress induction may have also induced stress specific changes in FC that are not related to rumination. Therefore, our FC results may be an entanglement of stress-specific and ruminative processes. On the other hand, the stress induction reliably induced state rumination in both groups and may be an ecologically more valid method for rumination induction than biographical induction methods (e.g., remembering a situation in which a subject ruminated the last time), since rumination usually occurs spontaneously and involuntary following certain internal and external triggers. Also, the used paradigm left the participants blind for the investigated process, which might prevent social desirability biases.

## Conclusions

In conclusion, we found higher baseline FC and reduced stress-induced FC reactivity within high ruminators. The FC reactivity was negatively associated with post-stress rumination. To the knowledge of the authors, this is the first study investigating the relationship of FC changes through social stress in high and low ruminators. The stress induction was reliably associated with different measurements of state rumination. The paradigm might be a promising tool to assess FC-related changes in clinical populations that are known to show stress-sensitive effects. In future studies, the passive assessment of state rumination over multiple FC measurements might give additional information about rumination-specific FC changes.

## Electronic supplementary material

Supplementary Material
