# Supplementary material for: Disrupted prefrontal functional connectivity during post-stress adaption in high ruminators
Source: Sci Rep. 2018 Oct 22;8:15588. doi: 10.1038/s41598-018-33777-1 (PMC6197217; doi:10.1038/s41598-018-33777-1)
Supplement: Supplementary file 1 — Supplementary Material [file 41598_2018_33777_MOESM1_ESM.docx]

**Disrupted prefrontal functional connectivity during post-stress adaption in high ruminators**

David Rosenbaum^1^, Paula Hilsendegen^1^, Mara Thomas^1^, Florian B. Haeussinger^1^, Hans-Christoph Nuerk^3,4,5^, Andreas J. Fallgatter^1,2,4^, Vanessa Nieratschker^1,2^, Ann-Christine Ehlis^1,4^, Florian G. Metzger^1,6^

^1^ Department of Psychiatry and Psychotherapy, University Hospital of Tuebingen, Tuebingen, Germany

^2^ Center of Integrative Neuroscience (CIN), Cluster of Excellence, University of Tuebingen, Germany

^3^ Department of Psychology, University of Tuebingen, Germany

^4^ LEAD Graduate School and Research Network, University of Tuebingen, Tuebingen, Germany

# ^5^ Leibniz-Institut für Wissensmedien, Tuebingen, Germany.

^6^ Geriatric Center, University Hospital Tuebingen, Tuebingen,Germany

**Supplemental Material**

State rumination was measured with the Amsterdam Resting-State Questionnaire with the following additional items from the RRS:

I thought about all my shortcomings, failings, faults and mistakes.

I thought about why I can’t handle things better.

I thought about why I have problems other people don’t have.

I thought about why I misbehaved in certain situations.

I thought about whereby I deserved my current life situation.

I couldn’t leave my negative thoughts aside.

I thought about past situations that I regret.

I thought about all my problems and worries.

Additionally a semi-structured interview about the ruminative habits has been assessed concerning the following dimensions:

Presence of dwelling thoughts

Persistence of ruminative content

Focus on past events

High personal relevance of thought content

Feelings of guilt, sham or sadness

Perceived hopelessness

Abstract processing as indicated by

Absence of behavioral actions

Absence of solutions

Non-concrete thought content

Why-questions

Duration of daily rumination

Felt impairments through rumination


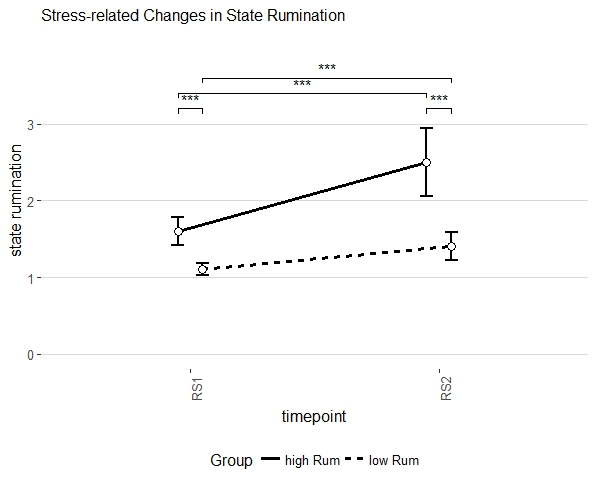
Figure S1. State rumination during resting state before and after the stress induction in the high (high Rum) and low (low Rum) trait ruminators. RS1 = pre-stress resting state, RS2 = post-stress rumination, *p<.05, **p <.01, ***p <.001.


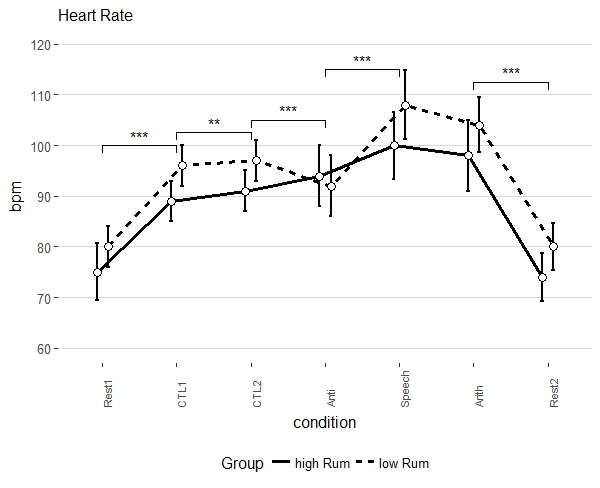


Figure S2. Heart rates during the different experimental conditions in the high (high Rum) and low (low Rum) trait ruminators. Rest1 = pre-stress resting state, CTL1 = control task 1/ reading numbers, CTL2 = control task 2/ performing subtractions without social stress, Anti = anticipatory stress phase, Speech = free speech challenge, Arith = arithmetic stress challenge, Rest2 = post-stress rumination, *p<.05, **p <.01, ***p <.001.


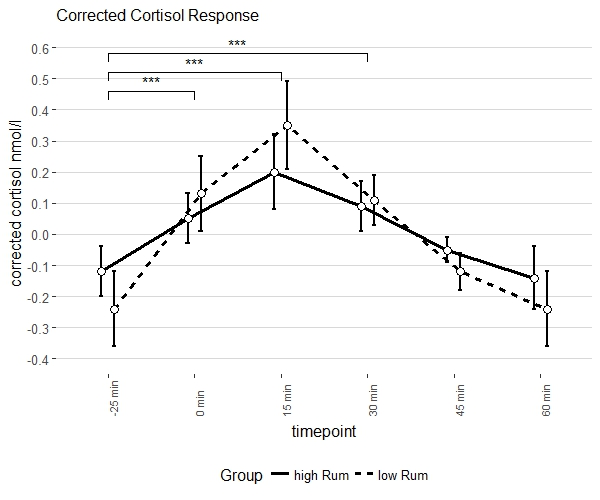


Figure S3. Corrected response in cortisol due to the TSST in the high (high Rum) and low (low Rum) trait ruminators. RS1 = pre-stress resting state, RS2 = post-stress rumination, *p<.05, **p <.01, ***p <.001.


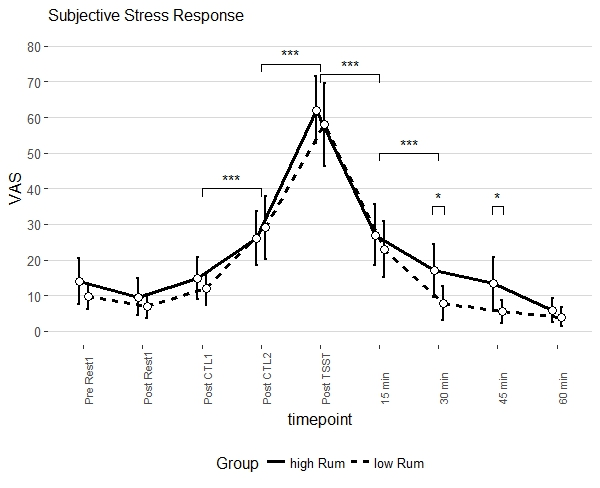


Figure S4. Subjective stress levels as assessed with visual analogue scales (VAS) during the experiment in the high (high Rum) and low (low Rum) trait ruminators. Pre Rest1 = before first resting state, Post Rest1 = after first resting state, CTL1 = control task 1/ reading numbers, CTL2 = control task 2/ performing subtractions without social stress, post TSST = after completion of the 16 min Trier Social Stress Test, *p<.05, **p <.01, ***p <.001.


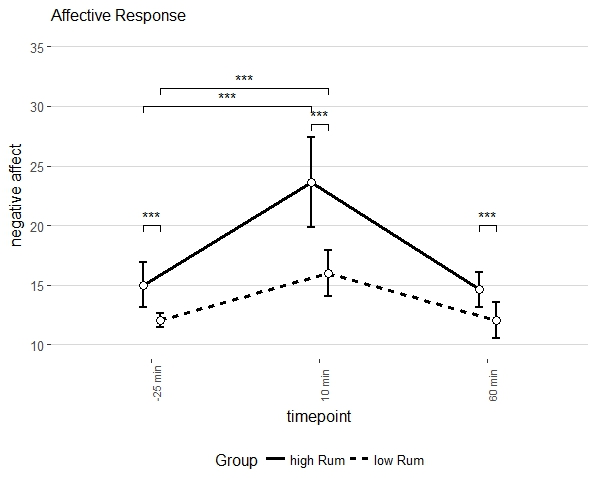


Figure S5. Negative affect before, 10 minutes after and one hour after the TSST in the high (high Rum) and low (low Rum) trait ruminators. *p<.05, **p <.01, ***p <.001.


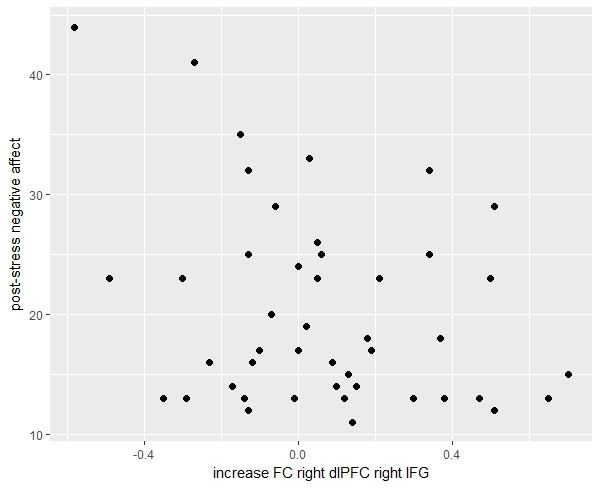


Figure S6. Correlation between post-stress negative affect and increases in functional connectivity of the right dorsolateral prefrontal cortex and right inferior frontal gyrus.


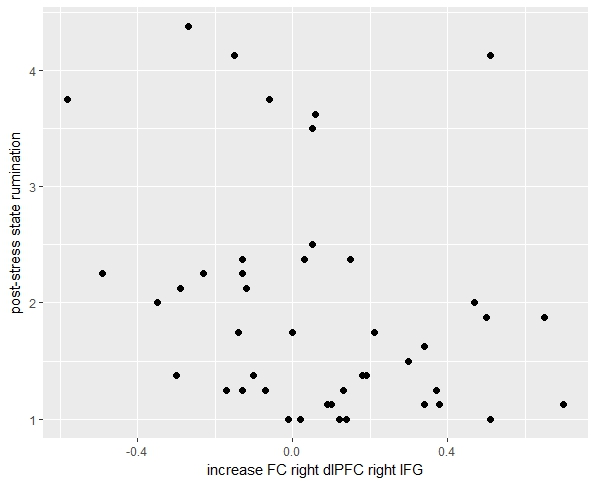


Figure S7. Correlation between post-stress state rumination and increases in functional connectivity of the right dorsolateral prefrontal cortex and right inferior frontal gyrus.
